# Indocyanine‐guided ureter resection for radical cystectomy – a systematic review and meta‐analysis

**DOI:** 10.1111/bju.16707

**Published:** 2025-03-25

**Authors:** Caelán Max Haney, Alexander Studier‐Fischer, Mark Enrik Geissler, Jakob Ohlmeier, Niklas Westhoff, Jens‐Uw Stolzenburg, Maurice Stephan Michel, Karl‐Friedrich Kowalewski

**Affiliations:** ^1^ Intelligent Systems and Robotics in Urology German Cancer Research Center (DKFZ) Heidelberg Germany; ^2^ DKFZ Hector Cancer Institute at the University Medical Center Mannheim Mannheim Germany; ^3^ Department of Urology and Urosurgery, University Medical Centre Mannheim University of Heidelberg Mannheim Germany; ^4^ Department of Visceral, Thoracic and Vascular Surgery, Faculty of Medicine and University Hospital Carl Gustav Carus TUD Dresden University of Technology Dresden Germany; ^5^ Department of Urology University of Leipzig Leipzig Germany

**Keywords:** bladder cancer, ureteric obstruction, radical cystectomy, indocyanine green, fluorescence imaging

## Abstract

**Objectives:**

To perform a systematic review and meta‐analysis of studies comparing indocyanine green (ICG)‐guided resection of ureters with the standard of care during radical cystectomy (RC).

**Methods:**

The Cochrane Central Register of Controlled Trials (CENTRAL), Medical Literature Analysis and Retrieval System Online (MEDLINE) and Web of Science were searched for studies comparing ICG‐guided resection of ureters with the standard of care during RC. The primary outcome was the rate of uretero‐intestinal stenosis (UIS) per patient, secondary outcomes included the rate of UIS per ureter, major and minor complications; re‐interventions due to UIS, re‐admissions and the length of ureter resected. Data were pooled as odds ratio (OR) or mean difference with a random‐effects model. Risk of bias was assessed using the Risk Of Bias In Non‐randomised Studies of Interventions (ROBINS‐I) tool and the Grading of Recommendations, Assessment, Development, and Evaluation (GRADE) approach was used to assess certainty of evidence. The systematic review was registered prospectively via the International Prospective Register of Systematic Reviews (PROSPERO: CRD42024545516).

**Results:**

In all, 11 studies totalling 1339 patients were identified. ICG‐guided resection led to a statistically significant decrease in UIS per patient (OR 0.20, 95% confidence interval [CI] 0.07–0.52) and per ureter (OR 0.17, 95% CI 0.06–0.50). There were statistically significantly fewer major complications, re‐interventions due to UIS in the ICG‐guided group, there was no difference in minor complications and re‐admissions. Certainty of evidence was low.

**Conclusions:**

With low certainty of evidence, ICG‐guided resection of ureters lowers the rate of UIS. A standardisation of grading of UIS is needed. The time for randomised controlled trials in this setting is now.

**Abbreviations**
CTCAE: Common Terminology Criteria for Adverse Events
GRADE: Grading of Recommendations, Assessment, Development, and Evaluation
ICG: indocyanine green
MD: mean difference
OR: odds ratio
PRISMA: Preferred Reporting Items for Systematic Reviews and Meta‐Analyses
(O)(RA)RC: (open) (robot‐assisted) radical cystectomy
ROBINS‐I: Risk Of Bias In Non‐randomised Studies of Interventions
UIS: uretero‐intestinal stenosis

## Introduction

Radical cystectomy (RC) is the mainstay of surgical treatment for muscle invasive bladder cancer [1]. Conventionally performed as open RC (ORC), robot‐assisted RC (RARC) has become an alternative and current evidence suggests that complications and long‐term outcomes are comparable [2]. During most RCs, a uretero–intestinal anastomosis must be made to create the urinary diversion. Depending on study and definition, uretero‐intestinal stenosis (UIS) affects about 10–15% of patients and represents a common short‐ but also longer‐term complication [3]. UIS can lead to kidney failure, recurrent UTIs and flank pain, necessitating insertion of either percutaneous drainage or ureteric stents. Simple endoscopic dilatation of the stenosis has shown to only be successful in the minority of cases. Thus, ureteric re‐implantation must be performed for a stent/nephrostomy‐free life [3, 4]. Due to the risk of complications when operating in a previously operated field, many patients opt for permanent percutaneous nephrostomy or ureteric stents. These patients regularly visit the outpatient clinic for material changes. In addition to planned visits, these patients have higher risks of UTIs due to the indwelling foreign bodies and frequently experience inadvertent removal of percutaneous drains or stents making visits to the emergency room necessary.

In the literature, the underlying reason for UIS is mostly cited to be insufficient blood supply of the distal end of the ureter [4, 5]. With the naked eye, visualising this insufficient supply is not possible. Therefore, it has been suggested that the use of indocyanine green (ICG), a fluorophore that emits light at wavelengths of 800 nm upon excitation and is used for various healthcare applications, could be used to visualise the ureters and their blood supply intraoperatively [6]. Recently, multiple studies examining this have been published in the literature.

The goal of this study was to perform a systematic review and meta‐analysis of studies comparing the use of ICG with no ICG for guidance of ureter resection during RC. The primary outcome was the rate of patients with UIS.

## Methods

This systematic review was performed in line with the Preferred Reporting Items for Systematic Reviews and Meta‐Analyses (PRISMA) statement and prospectively registered via the International Prospective Register of Systematic Reviews (PROSPERO CRD42024545516). For easier evaluation of the review, the A MeaSurement Tool to Assess systematic Reviews (AMSTAR)‐2 critical appraisal tool has been completed and is available in Data S1.

### Eligibility Criteria and Search Strategy

A database search via the Medical Literature Analysis and Retrieval System Online (MEDLINE, via PubMed), Web of Science and Cochrane Central Register of Controlled Trials (CENTRAL) was performed using the listed ‘PICO’ criteria until 19 April 2024. Additional trials were identified via a keyword search (‘radical cystectomy’, ‘indocyanine green’, ‘ureteroenteric stricture’) via Google Scholar up to 7 May 2024. There were no restrictions regarding language.

P (patients): patients undergoing RC

I (intervention: resection of the ureters based on ICG

C (control): resection of the ureters based on clinical judgement

O (outcome): ureteroenteric strictures

S (study design): comparative studies

The search strategies are listed in the Data S1.

### Study Selection

Initially, a title and abstract screening was performed to screen for studies that could fit the criteria. To heighten the sensitivity, when studies mentioned ICG and RC, they were included in the full‐text screening, unless they explicitly did not fit the criteria (e.g., case reports). Systematic reviews and narrative reviews with the subject of ICG and urology were included in the full‐text screening with the goal of identifying potential studies in the references. After initial screening, full texts were acquired and screened for inclusion.

The entire process was performed by two reviewers independently, conflicts were solved by a third reviewer.

### Data Extraction

Data were extracted by two reviewers independently using a pre‐specified, piloted Excel sheet. Data on study characteristics (e.g., authors, year, country, multicentric, retrospective), patients (e.g., age, body mass index, tumour characteristics, neoadjuvant chemotherapy), operating technique (RARC, intracorporeal urinary diversion, anastomotic technique), and outcomes were collected. In case of conflict, a third reviewer assessed the data. In unclear cases, the study authors were contacted for clarification of data.

### Outcomes

The primary outcome was the rate of UIS per patient as reported by the studies. Further outcomes were the rate of UIS per ureter, complications (Clavien–Dindo Grade ≥III; Clavien–Dindo Grade <III); re‐interventions due to UIS, re‐admissions, length of ureter resection, large segment resections, and UTIs. Exact definitions of the outcomes are presented in the methods section of the Data S1.

### Risk of Bias and Certainty of Evidence Assessment

Risk of bias was assessed using the Risk Of Bias In Non‐randomised Studies of Interventions (ROBINS‐I) tool according to the relevant guidelines. Pre‐specified confounding domain was obesity and pre‐specified relevant co‐interventions were switches from interrupted to continuous sutures for uretero–intestinal anastomosis, switches between ORC and RARC, and a switch from intracorporeal to extracorporeal anastomosis technique. Certainty of evidence was assessed with the help of the Grading of Recommendations, Assessment, Development, and Evaluation (GRADE) tool. Further details are on risk of bias and certainty of evidence are described in the Data S1.

### Statistical Analysis

Data analysis was performed in line with the Cochrane Handbook of Systematic Reviews and Interventions and the recommendations of the Study Center of the German Society of Surgery (Kalkum, 2021 #33). Forrest plots are used for presentation of the odds ratio (OR) and 95% CIs which were calculated by Mantel–Haenszel model for dichotomous data and mean difference (MD) with CI for continuous data, which was calculated using the inverse variance model. A random‐effects model was used for the calculations and heterogeneity was reported with the help of *I* ^2^. Publication bias was assessed with funnel plots using the primary outcome.

## Results

**Fig. 1:**
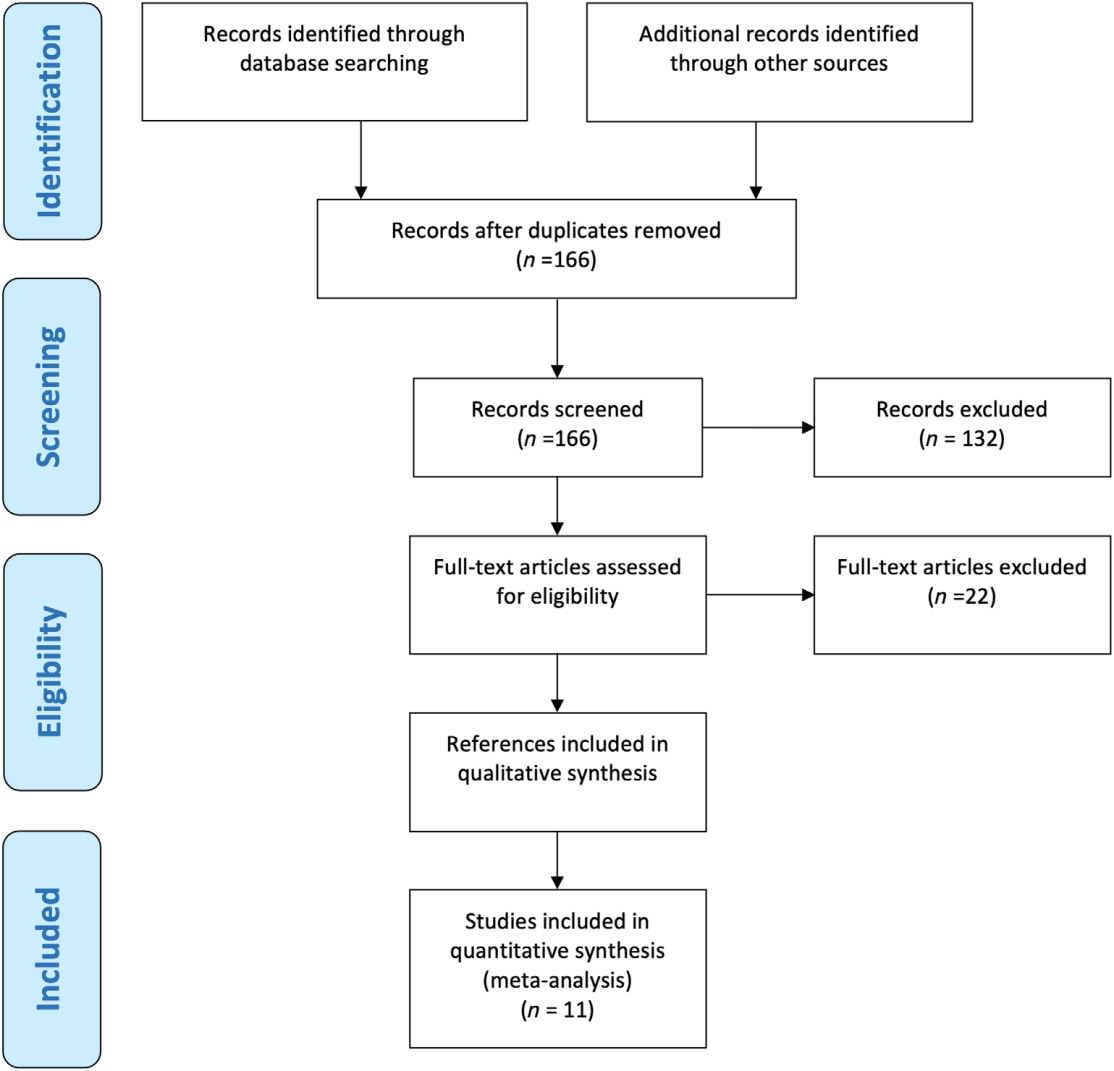
The PRISMA flow chart.

The systematic search via Web of Science, MEDLINE and CENTRAL identified 159 relevant studies. After title and abstract screening, 27 references were full‐text screened, of which five fitted the inclusion criteria. Through the additional non‐systematic search via Google Scholar, seven further studies were identified, which fitted the inclusion criteria. Of these, four were congress abstracts, one study was published in Russian, and one journal was not listed in PubMed. The last study was not found via the search because it did not include ‘radical cystectomy’ in title or abstract but only ‘neobladder formation’. Two congress abstracts (Carbonell et al. [7] and Font et al. [8]) reported data from the same cohort, here, the larger study was used unless data were only reported in one abstract. Overall, 11 studies including 1339 patients were included in the systematic review of which 10 provided data on the primary outcome (Fig. 1) [7, 8, 9, 10, 11, 12, 13, 14, 15, 16, 17].

In all, 10 studies were completely retrospective cohort studies, one study prospectively assessed ICG‐guided ureter resection and compared this with a retrospective control cohort. The study characteristics and patient characteristics are presented in Table 1 [7, 8, 9, 10, 11, 12, 13, 14, 15, 16, 17; 18], additional details are listed in Table S1.

**Table 1:** Baseline characteristics.

Reference	Approach	Number of patients		Age, years		BMI, kg/m^2^		Organ confined, %		Node positive, %		Neoadjuvant chemotherapy, %		Ileum conduit, %		Follow‐up, months		Side effects of ICG
		ICG	No‐ICG	ICG	No‐ICG	ICG	No‐ICG	ICG	No‐ICG	ICG	No‐ICG	ICG	No‐ICG	ICG	No‐ICG	ICG	No‐ICG	
Ahmadi et al., 2019 [9]	RARC	47	132	68 (44–92)^‡^	72.4 (45–89)^‡^	27 (15–32)^‡^	27 (19–44)^‡^	51	48	32	32	23	24	68	73	12 (2–20)^‡^	14 (1–42)^‡^	None
Carbonell et al. [7] and Font et al., 2024 [8]	RARC	79	142	69 (63–74)*		–	–	–	–	–	–	24	39	na	na	21.1 (10.5‐35.2)*		NR
Doshi et al., 2020 [10]	ORC	31	30	67.5 (59.2–73.5)*	71.6 (64.9–74.4)*	28.3 (25.4–32.2)	29.4 (26.4–32)	–	–	–	–	52	53	71	72	15.8 (12.2‐18.1)*	23.2 (7–29.3)*	None
Fu et al., 2024 [11]	RARC	138	100	67 (59–74)*	64 (57–75)*	–	–	–	–	–	–	–	–	37	19	–	–	NR
Narita et al., 2021 [12]	RARC	18	31	–	–	–	–	–	–	–	–	–	–	42	59	–	–	NR
Pavlov et al., 2023 [13]	RARC	22	34	66 (5.2)^†^	69 (4.5)^†^	27.1 (2.5)^†^	26.5 (3.8)^†^	–	–	–	–	–	–	–	–	14	12	None
Petrut et al., 2021 [14]	RARC, LRC	27	28	–	–	–	–	–	–	–	–	–	–	0	0	11	–	None
Shen et al., 2019 [15]	RARC	47	47	69 (65–76)*	74 (62–80)*	24.7 (23.9–30.3)*	25.9 (24.6–28.8)*	–	–	–	–	–	–	55	60	12	24.3	None
Tuna et al., 2022 [16]	RARC	10	7	69 (67–78)*	70.5 (65–73)*	29 (28–30)*	28.5 (27–31)*	29	80	0	20	29	30	86	40	–	–	NR
Wang et al., 2022 [17]	RARC	20	25	–	–	–	–	–	–	–	–	–	–	100	100	8	19	NR
Yeaman et al., 2024 [18]	ORC	55	277	66.3 (10.6)^†^	65.6 (10.4)^†^	28.2 (5.3)^†^	28.5 (6.8)^†^	–	–	–	–	–	–	98	94	17.5	58	None

In ‘Side effects of ICG’, ‘None’ indicates that the publication explicitly reported that there were no associated complications, whereas ‘NR’ indicates that the publication did not report whether there were any complications. BMI, body mass index; LRC, laparoscopic radical cystectomy; NR, not reported.

* Median (interquartile range).

^†^ Mean (standard deviation).

^‡^ Median (range).

### Primary Outcome

#### Uretero‐intestinal Stenoses Per Patient

**Fig. 2:**
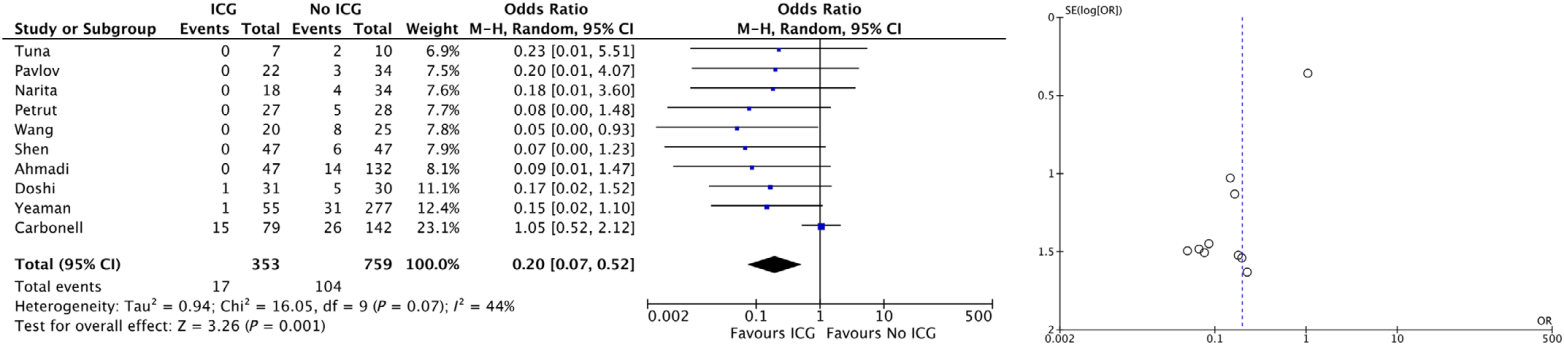
Results of the meta‐analysis for the primary outcome: strictures per patient (left) and the corresponding funnel plot (right), indicating a potential small study publication bias.

In all, 10 studies totalling 1112 patients were included in the meta‐analysis. With low certainty of evidence, there was a strong effect of ICG‐guided resection of ureters during RC in lowering the rate of patients with UIS (OR 0.20, 95% CI 0.07–0.52; Fig. 2). Limiting the evidence was that most studies did not report an explicit definition of UIS or their exact follow‐up protocol, and protocol in case of suspicion of UIS. The management of UIS was also not explicitly described in most studies, so that this was a source of heterogeneity. The effect reached the pre‐defined limit of a strong effect with an OR of <0.5 and a CI not crossing the margin of minimum effect. Additionally, heterogeneity as measured by *I* ^2^ was moderate and mostly relied on one study that showed no effect. Upon visual assessment of the funnel plot, there was potential evidence of a small study publication bias, with small studies without an effect being disadvantaged in publication.

### Secondary Outcomes

#### Uretero‐intestinal Stenoses Per Ureter

**Fig. 3:**
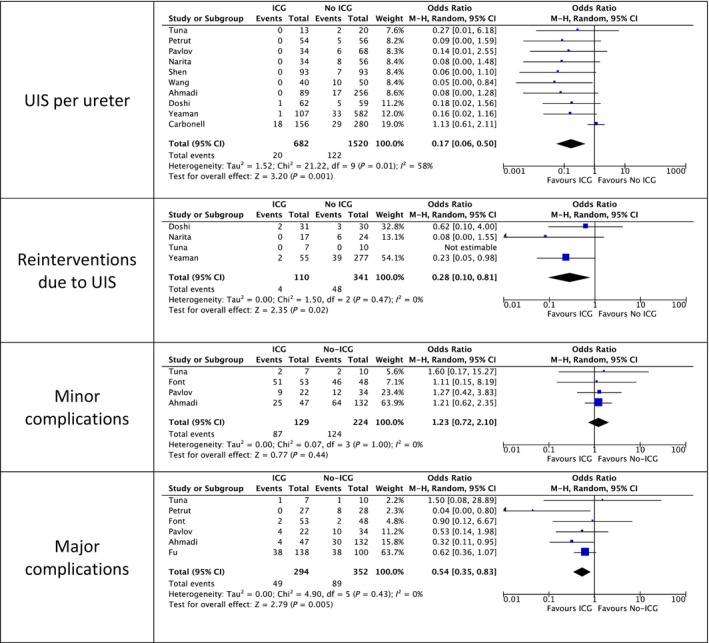
Meta‐analysis results of the secondary outcomes.

In all, 10 studies totalling 2202 ureters were included in the meta‐analysis. With low certainty of evidence, there was a strong effect of ICG‐guided resection of ureters during RC in lowering the rate of patients with UIS (OR 0.17, 95% CI 0.06–0.50; Fig. 3). There were similar limitations as with UIS per patient.

#### Complications

Six studies totaling 646 patients reported Clavien‐Dindo Grade ≥III complications and were included in the meta‐analysis. With low certainty of evidence, there was a moderate effect of ICG‐guided resection of ureters during RC in lowering the rate of serious complications (OR 0.54, 95% CI 0.35–0.83; Fig. 3).

With only four studies totaling 353 patients reporting Clavien‐Dindo Grade <III complications, there was no statistically significant difference between the groups (OR 1.23, 95% CI 0.72–2.10; Fig. 3).

#### Re‐interventions Due to UIS

Four studies with 451 patients reported re‐interventions due to UIS. There were significantly fewer re‐interventions due to UIS in the ICG‐guided group (OR 0.28, 95% CI 0.10–0.81; Fig. 3). Limiting the evidence was that not all studies adequately reported if all patients with UIS underwent a re‐intervention (e.g., ‘If there is high index of suspicion of stricture (no reflux on loopogram/cystogram, and/or rising creatinine, and/or worsening hydronephrosis), we usually place a percutaneous nephrostomy tube and obtain a confirmatory antegrade nephrostogram’. [9]).

#### Re‐admissions

Only one study with 179 patients reported the rate of re‐admissions. There was no statistically significant difference between the two groups (OR 1.28, 95% CI 0.64–2.57).

#### Urinary Tract Infections

No studies reported the rate of UTIs.

#### Excised Ureter Length

Four studies totaling 942 ureters reported the length of resected ureter. There was a small statistically significant difference in length of resected ureter with more ureter resected in the ICG‐guided group (MD 2.51 mm, 95% CI 0.93–4.08 mm).

Regarding resections of large segments of ureter (≥5 cm), there was a statistically significant difference with more events in the ICG‐guided group, two studies with 447 ureters reported data (OR 3.29, 95% CI 1.72–6.27). Data is presented in the Data S1 (Fig. S1).

### Risk of Bias

Risk of bias for the main outcome as assessed with the ROBINS‐I tool was severe. The intervention group was retrospective in all but one study, the control group was retrospective in all studies. Most studies did not justify why they chose the timepoints for assessment, the definition of UIS varied strongly across studies. No study adjusted for any risk factors. One study had multiple co‐interventions as it reported on a quality improvement programme. This led to a mostly severe rating of risk of bias. The risk of bias assessment is portrayed in the Table S2 and Fig. S2.

### Certainty of Evidence

Certainty of evidence per GRADE is demonstrated in Table 2. The main reasons for downgrading were the retrospective nature of the studies resulting in a high risk of bias rating. Additionally, upgrading of certainty of evidence was performed due to a very strong effect for UIS, while all outcomes were downgraded due to the risk of publication bias.

**Table 2:** Certainty of evidence table as assessed via GRADE.

ICG‐guided resection of ureters compared to standard of care for RC						
Outcomes	Anticipated absolute effects* (95% CI)		Relative effect (95% CI)	Number of participants (studies)	Certainty of the evidence (GRADE)	Comments
	Risk with standard of care	Risk with ICG				
UIS (per patient)	137 per 1000	31 per 1000 (11–76)	OR 0.20 (0.07–0.52)	1112 (10 non‐randomised studies)	⨁⨁◯◯ Low^a^, ^b^	With low certainty of evidence, ICG‐guided ureter resection leads to a strong decrease in patients with UIS in patients undergoing RC
UIS (per ureter)	80 per 1000	15 per 1000 (5–42)	OR 0.17 (0.06–0.50)	2202 (10 non‐randomised studies)	⨁⨁◯◯ Low^a^, ^b^	With low certainty of evidence, ICG‐guided ureter resection leads to a strong decrease in ureters with UIS in patients undergoing RC
Re‐interventions due to strictures	141 per 1000	44 per 1000 (16–117)	OR 0.28 (0.10–0.81)	451 (4 non‐randomised studies)	⨁◯◯◯ Very low^a^, ^b^, ^c^	With very low certainty of evidence, ICG‐guided ureter resection leads to a significant reduction of re‐interventions due to strictures in patients undergoing RC
Clavien‐Dindo ≥III complications	253 per 1000	155 per 1000 (106–219)	OR 0.54 (0.35–0.83)	646 (6 non‐randomised studies)	⨁◯◯◯ Very low^a^, ^b^	With very low certainty of evidence, ICG‐guided ureter resection leads to fewer severe complications in patients undergoing RC
Clavien‐Dindo <III complications	554 per 1000	604 per 1000 (472–723)	OR 1.23 (0.72–2.10)	353 (4 non‐randomised studies)	⨁◯◯◯ Very low^a^, ^b^, ^d^	There is no certain evidence on whether ICG‐guided ureter resection leads to an increase or decrease of minor complications
Re‐admissions	326 per 1000	382 per 1000 (236–554)	OR 1.28 (0.64–2.57)	179 (1 non‐randomised study)	⨁◯◯◯ Very low^a^, ^b^, ^c^, ^d^	There is no certain evidence on whether ICG‐guided ureter resection leads to an increase or decrease of readmissions due to UIS

^a^ Severe risk of bias as assessed with ROBINS‐I tool;

^b^ Likely small studies with no to negative effects not published;

^c^ Optimal information size for hypothetical trial not reached;

^d^ Large CI.

* The risk in the intervention group (and its 95% CI) is based on the assumed risk in the comparison group and the relative effect of the intervention (and its 95% CI).

GRADE Working Group grades of evidence

High certainty: we are very confident that the true effect lies close to that of the estimate of the effect.

Moderate certainty: we are moderately confident in the effect estimate: the true effect is likely to be close to the estimate of the effect, but there is a possibility that it is substantially different.

Low certainty: our confidence in the effect estimate is limited: the true effect may be substantially different from the estimate of the effect.

Very low certainty: we have very little confidence in the effect estimate: the true effect is likely to be substantially different from the estimate of effect.

## Discussion

This study presents the first systematic review and meta‐analysis to compare ICG‐guided resection of ureters with conventional resection guided by clinical evaluation for RC.

In all, 11 studies totalling 1339 patients were included, with patients undergoing both ORC and minimally invasive RC and receiving both ileum conduit and neobladders.

Overall, there was a strong and clinically relevant decrease in UIS per patient benefitting the ICG‐guided resection. This benefit was confirmed in the evaluation per ureter. Additionally, there was a difference in major complications favouring the ICG‐guided group. There were no statistically significant differences in re‐interventions, re‐admissions, or minor complications. The certainty of evidence as assessed with GRADE was low.

Uretero‐intestinal stenosis represents one of the major both short‐ and longer‐term complications after RC. While the majority of UIS occur within the first year, benign UIS can occur up to 5 years after RC [19]. Risk factors that have been identified are obesity, the use of continuous sutures for the uretero–intestinal anastomosis, and a complicated postoperative course. Most recently, a large systematic review including >3000 patients identified RARC as a potential risk factor [20]. However, it is currently unclear if this increase in risk is due to the initial learning curve of RARC or due to other underlying reasons, such as more extensive dissection of the ureters when performing extracorporeal urinary diversion.

To date, ischaemia as the underlying aetiology of UIS had not yet been confirmed. This meta‐analysis mechanistically supports ischaemia as the aetiology as resecting segments of the ureter that did not appear perfused led to fewer UIS.

Importantly, the evidence here applies both for minimally invasive RC and ORC. While the majority of studies used the Firefly™ system, which is integrated into the da Vinci^®^ surgical system (Intuitive Surgical Inc., Sunnyvale, CA, USA), three studies used the SPY^®^ fluorescence imaging system by Stryker AB (Malmö, Sweden). In a sensitivity analysis, the results did not change significantly when using either system.

Important questions remain regarding the optimal timepoint, technique and dosage for ICG assessment. Most studies describe the timepoint of assessment to be after resection of the ileum and directly before implantation of the ureters into the intestine, while in one study, the assessment took place before resection of the ileum [15]. The authors justify this choice with being able to resect a longer piece of ileum if necessary. While this appears logical, none of the other studies mention that choosing a different timepoint led to problems in construction of the anastomosis and two studies explicitly describe that there were no changes in choice of urinary diversion due to the results of ICG inspection. Even though not reported, it seems possible that the resection of a large segment of ureter could also lead to a change in anastomotic strategy from Wallace to Bricker if the ureters are not long enough to be joined together. However, to date no significant differences between the two anastomotic techniques have been shown, so that this would likely not be a problem. One study also performed a second assessment after the creation of the uretero–intestinal anastomosis. However, they did not report if this led to any significant treatment changes.

The dosage of ICG used across the included studies varied between 5 and 25 mg, reflecting the absence of a standardised protocol for its administration during RC. While the precise impact of dosage on ureter visualisation and perfusion assessment remains unclear, it is plausible that higher doses could enhance fluorescence intensity and prolong the duration of visualisation. However, excessive dosing may also lead to non‐specific background fluorescence, potentially reducing contrast and impairing the accuracy of ischaemia assessment. None of the included studies specifically analysed the relationship between ICG dose and the incidence of UIS, making it difficult to draw definitive conclusions on dose‐dependent effects.

Regarding further aspects of the technique of ICG usage, the recently published consensus conference statement on fluorescence‐guided surgery by the European Society of Surgical Oncology has recommended that haemodynamic variables, such as blood pressure, should be considered when evaluating the fluorescent signal [21]. Low blood pressure with resulting malperfusion of distant capillaries could lead to the perfusion of the ureters appearing worse than actually the case. Especially in operations in which both ureters appear significantly malperfused, blood pressure should be checked, and in case of low blood pressure, ICG assessment should be re‐performed after normalisation. Also to be kept in mind should be that the application of ICG before ureteric dissection during surgery may result in background fluorescence, potentially leading to false‐positive signals in ischaemic ureteric segments. This risk could arise if the ileal segment is prepared before ureteric dissection is complete and ICG is used to assess the ileal anastomosis, or in the context of sentinel lymph node dissection. These factors should be carefully considered when combining these techniques to avoid misinterpretation of ureteric perfusion.

Regarding complications due to the use of ICG, no anaphylactic reactions or similar events were reported by the included studies. Only five studies explicitly report that there were no complications related to ICG use, while there was no discussion of ICG‐related complications in the other studies. While there are reports of anaphylactic reactions in the literature, these are not reported to be very common [22].

### Limitations

One major limitation of the presented systematic review and meta‐analysis is the lack of clear definition of what constitutes a UIS. This is likely the reason for the strong heterogeneity in UIS rates, which varied between 0% and 19% in the intervention group, and 9% and 32% in the control group. From our knowledge, there is no currently accepted definition that is widely used in the literature. As such, many of the include studies did not adequately describe their definition of UIS. This ranged from no description at all, to inadequately described definitions such as ‘signs of stricture formation were present (absence of reflux on pyelogram and/or elevated creatinine level and/or progression of hydronephrosis)’ that leave room for interpretation and might bias results [13]. The most common definition of UIS included some kind of advanced imaging (e.g., loopogram/cystogram with lack of reflux; diuretic renal scan; CT scan with focal narrowing and related hydronephrosis), which were triggered by blood work findings (e.g., elevated serum creatinine; elevated infectious parameters), symptoms or routine imaging (e.g., ultrasound). However, many of these definitions result in a very heterogenous group of patients with varying degrees of symptoms and varying degrees of severity and do not provide a level of granularity that will enable comparing results over different groups and studies. As such, cases that are very common in clinical practice such as patients presenting without symptoms to a routine 3‐month check‐up with slight (possibly intermittent) hydronephrosis after RC with an ileum conduit might be classified as having stenosis in one study, while a patient having to undergo temporary percutaneous nephrostomy during a UTI after RC but presenting without focal narrowing in CT might be classified as not having UIS in another study.

Additionally, what might be more important than a concrete definition of UIS is the comparability of the complications related to UIS. The Common Terminology Criteria for Adverse Events (CTCAE) version 5.0, which was released in 2017, presents three categories into which complications related to UIS could be categorised. The terms include ‘urostomy obstruction’ (‘A disorder characterized by the blockage of the urostomy’), ‘urostomy stenosis’ (‘A finding of a narrowing of the opening of a urostomy’) and ‘urinary tract obstruction’ (‘A disorder characterized by blockage of the normal flow of contents of the urinary tract’). The CTCAE clusters severity of the adverse event into five grades, ending with Grade 5, which is defined as death due to the adverse event. The three definitions have in common that Grade 4 includes the need for urgent (operative) intervention due to life threatening consequences. However, the definition of Grade 4 for ‘urostomy obstruction’ also includes ‘organ failure’. This would result in many complications due to UIS being classified as Grade 4 due to the common finding of kidney failure, which, while definitively indicating higher severity, is not necessarily always immediately life threatening. Grade 3 presents more heterogeneously than the other grades, as there are some key differences. The definition for ‘urostomy obstruction’ includes the finding of sepsis while both ‘urostomy stenosis’ and ‘urinary tract obstruction’ do not. All definitions call for elective (operative) interventions and include the finding of hydronephrosis. It is unclear when the presence of sepsis due to ‘urostomy obstruction’ would not indicate urgent intervention, thus calling into question the validity of this grade. Grade 2 and 1 would likely not be used for most patients with UIS, as these most commonly present with some sort of hydronephrosis or (slight) renal dysfunction, which would automatically move them to Grade 3. When assessing these three definitions, while not yet perfect, the most accurate would likely be ‘urostomy stenosis’. While actually referring to the narrowing of the opening of a urostomy this could easily be transferred to a narrowing of the entire neourinary tract and resulting adverse events. However, this definition would classify two groups of patients into Group 3, namely patients requiring non‐urgent intervention and patients with hydronephrosis, even if these do not require intervention and do not have kidney dysfunction. Additionally, uncommon but severe adverse events such as the loss of a kidney unit due to chronic unnoticed UIS with resulting hydronephrosis go unrecognised in this classification.

#### Lack of Study Quality

A further limitation of this systematic review and meta‐analysis is that all but one study were performed retrospectively. This leads to a lack in quality with many patients likely not undergoing regular evaluations. However, the effect that was shown was consistent across most studies with only one study not showing an effect. When leaving this study out of the analysis, the heterogeneity dropped from 37% to 0%. Unfortunately, this was a congress abstract, which is why the underlying reason for the lack of effect was not able to be elucidated. It is possible that there was a difference in ICG usage or UIS definition. The only prospective study also included a retrospective component as it compared the prospective evaluation of ICG with a historical cohort. In most studies, the follow‐up in the ICG‐guided group was shorter than in the no‐ICG group; however, most of the time the follow‐up was >12 months, significantly crossing the median time to stenosis reported in the literature [3].

### Strengths

This is the first systematic review to assess this field of research. While multiple significant limitations were identified, the effect shown is consistent across studies and clinically highly relevant. It is even possible that the actual effect on patients might be underrepresented by this analysis, as the effect shown here only compares the number of UIS between the two groups. Oftentimes, patients experience multiple complications and treatments arising from UIS such as ureteric re‐implantation, re‐interventions due to stent occlusion or displacement, and frequent hospital and emergency room visits due to UTIs, which are likely underreported in these studies. While there are barely any data on this topic in the published literature, quality of life for patients with UIS is likely to be significantly lower than with comparable patients without UIS.

## Conclusion

It is likely that the use of intravenous ICG to guide the resection of ureters for patients undergoing RC lowers the rate of UIS and associated complications. No ICG associated complications were reported by the studies. A standardisation of the definition of UIS and complications due to UIS is needed to make results more comparable between centres and studies. The time for randomised controlled trials examining this approach is now.

## Disclosure of Interests

No authors report any relevant conflicts of interest regarding the manuscript.

## Supporting information


**Data S1.** Methods.
**Table S1.** Study characteristics.
**Table S2.** Risk of bias assessment.
**Fig. S1.** Ureter resection lengths.
**Fig. S2.** Risk of bias scoring.
