# Supplementary material for: Indocyanine‐guided ureter resection for radical cystectomy – a systematic review and meta‐analysis
Source: BJU Int. 2025 Mar 25;135(6):908–17. doi: 10.1111/bju.16707 (PMC12053116; doi:10.1111/bju.16707)

# Supplementary Material:

## Methods

### Search strategy

PUBMED:


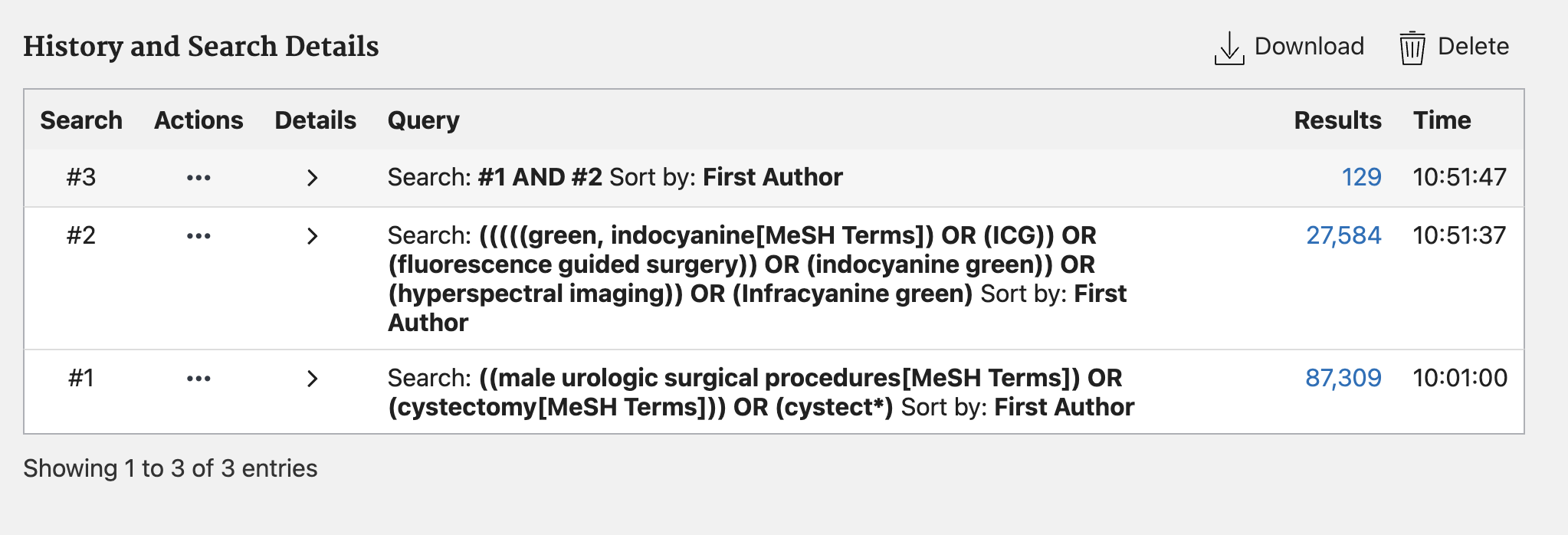


CENTRAL:


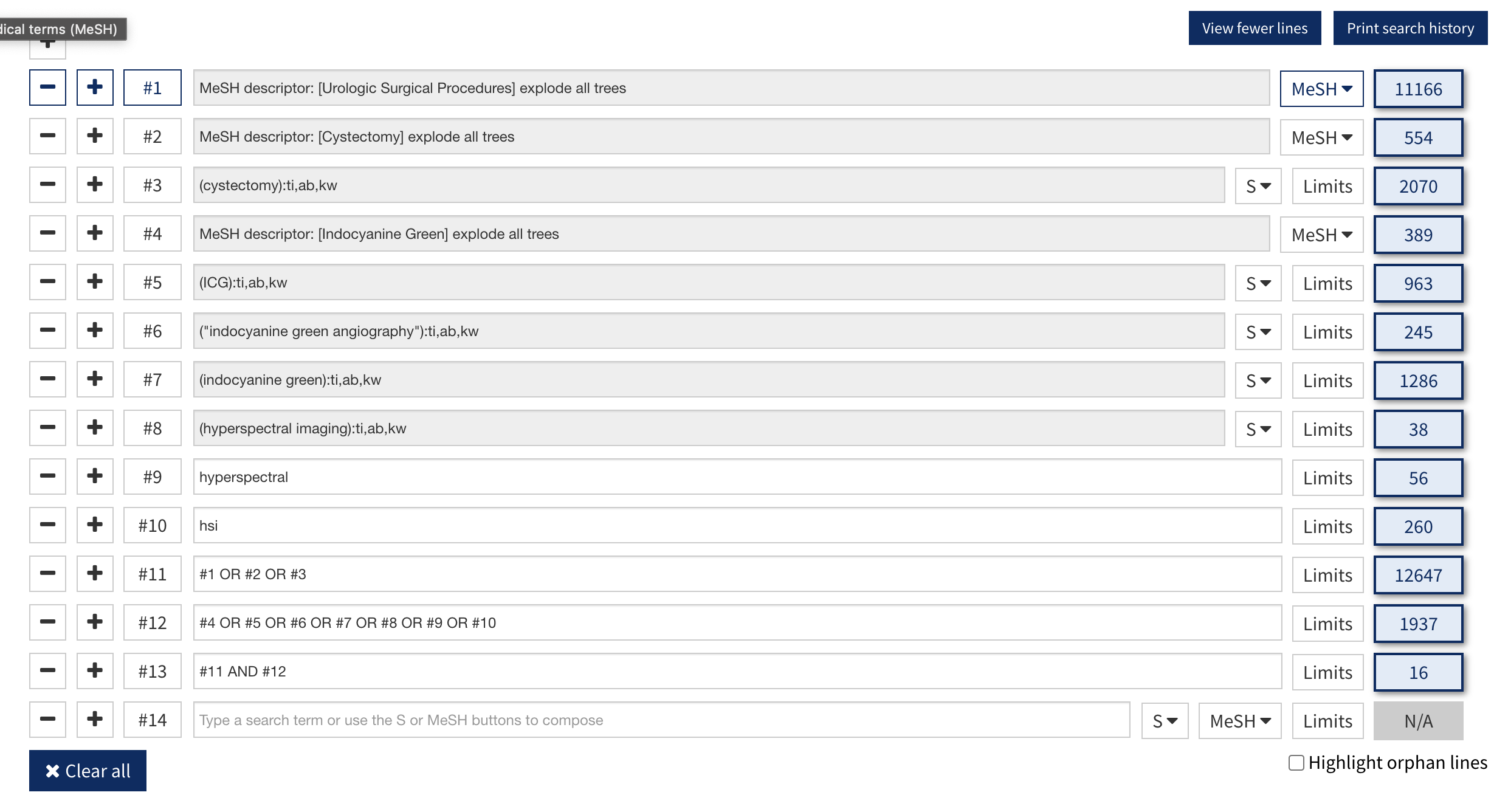


Web of Science:


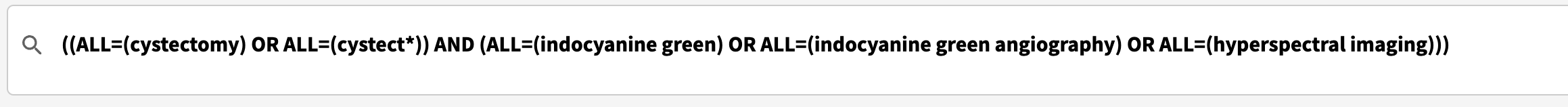


### Primary outcome:

The primary outcome was the rate of UIS per patient as reported by the studies. If studies did not clearly report the rates of UIS but reported hydronephrosis ≥ grade 2, this was used as a surrogate for the rate of UIS. If studies did not report the rate of UIS per patient but only per ureter, the rate of UIS per patient was calculated if there was data on the laterality of the stenoses and the number of patients with bilateral stenosis. If there was only data on the laterality of stenoses but not on the number of patients with bilateral stenosis, the rate was calculated by adding 50% of the number of right sided stenoses to the number of left sided stenoses, i.e. it was assumed that a certain number of patients suffered from bilateral stenoses. E.g. 20/150 UIS on left side, 10/150 UIS on right side, 35 patients: 20 + 5 = 25 patients with stenosis out of 150 patients.

### Secondary outcomes:

#### Ureterointestinal stenosis per ureter:

The definition of stenosis was as reported by the studies. This was also split up by laterality.

#### Complications:

Complications up to 90 days were rated by Clavien-Dindo and split up into high-grade (≥3) and low-grade (≤2).

#### Reinterventions due to UIS:

A reintervention due to UIS was defined to be every placement or change of ureteral stents or nephrostomies or reoperation for ureter reimplantation. Removal of stents was not defined as an intervention.

#### Readmissions:

These were calculated as reported by the individual studies.

#### Length of excised ureter:

This was measured in millimeters (mm) and split up by laterality.

#### Excised large segment:

Hereby, a large segment of ureter was defined to be more than 3 cm or more than 5 cm, and the data was split up by laterality.

#### Urinary tract infections:

These were calculated as reported by the individual studies.

### Risk of bias and certainty of evidence assessment:

Risk of bias was assessed using the ROBINS-I tool according to the relevant guidelines. The ROBINS-I tool defines confounding domains as pre-interventional prognostic factors that predict if a patient undergoes the intervention of interest, in this case resection of the ureters with the help of ICG. Plausibly speaking, these factors would be patient characteristics that put the patient at a high risk of stenosis. From a recent systematic review of risk factors for UIS after radical cystectomy obesity, RARC, postoperative complications and urinary leaks were identified. Of these, obesity is the only pre-interventional prognostic factor for which a realistic adjustment could be performed. ^7^ Therefore, obesity was prospectively defined as a confounding domain with lower weight indicating a lower risk for stenosis, benefitting the group with lower weight. If studies did not adjust for weight, a difference of 5 BMI points was chosen as the minimum significant difference indicating relevant confounding.

Relevant co-interventions that could be different between the groups were prospectively defined. These were: switch from interrupted to continuous sutures, switch between open and robotic-assisted surgery and switch from intracoporeal to extracorporeal technique. The co-interventions were chosen based on a scoping review of interventions that can influence the frequency of UIS. ^8 9^ All switches in technique as described in the directions above are judged to be associated with an increased risk of stenosis and vice versa. Certainty of evidence was assessed with the help of the GRADE tool. The minimum relevant effect was prospectively defined as an odds ratio of 0.8, a large magnitude of effect was prospectively defined as an odds ratio of less than 0.5, with the confidence interval not crossing the border of the minimum relevant effect. All assessments were performed by two individual raters independently, in cases of conflict a third rater was involved.

As an indicator of technique, a lack of difference in the length of excised ureter was defined.

## Results

### Included studies

#### Supplementary Table 1: Study Characteristics

| Study | Year published | Congress abstract/published study | Prospective / retrospective | beginning observation | end observation | ORC/RARC/LRC |
| --- | --- | --- | --- | --- | --- | --- |
| Ahmadi | 2019 | Published study | retrospective | 2014 | 2017 | RARC |
| Carbonell/Font | 2024 | Congress abstract(s) | retrospective | 2018 | 2023 | RARC |
| Doshi | 2020 | Published study | retrospective | 2017 | 2019 | ORC |
| Fu | 2024 | Published study | retrospective | 2012 | 2021 | RARC |
| Narita | 2021 | Published study | retrospective | 2013 | 2021 | RARC |
| Pavlov | 2023 | Published study | retrospective | 2021 | 2022 | RARC |
| Petrut | 2021 | Published study | retrospective | unclear | unclear | RARC, LRC |
| Shen | 2019 | Published study | retrospective | 2015 | 2018 | RARC |
| Tuna | 2022 | Published study | retrospective | 2014 | 2021 | RARC |
| Wang | 2022 | Congress abstract | retrospective | 2015 | 2020 | RARC |
| Yeaman | 2024 | Published study | Prospective (ICG)/retrospective (No-ICG) | 2021 (ICG); 2016 (No-ICG) | 2022 (ICG); 2021 (No-ICG) | ORC |

Table 1: Study characteristics (ORC = open radical cystectomy; RARC = robotic-assisted radical cystectomy, LRC = laparoscopic radical cystectomy)

### Outcomes

#### Supplementary Figure 1: Ureter resection lengths

| Length of ureter resected (mm) |  |
| --- | --- |
| Length of ureter resected (right ureter; mm) |  |
| Length of ureter resected (left ureter; mm) |  |
| Long segment of ureter resected (≥5 cm) |  |

### Risk of bias assessment

#### Supplementary table 2: Risk of bias assessment

| Study | Confounding | Selection of participants | Classification of intervention | Deviations from intervention | Missing data | Measurement of result | Selection of results | Overall |
| --- | --- | --- | --- | --- | --- | --- | --- | --- |
| Ahmadi | Serious | Low | Low | Low | Moderate | Serious | Serious | Serious |
| Carbonell | Serious | Low | Serious | Serious | Serious | Serious | Moderate | Serious |
| Doshi | Serious | Low | Low | Low | Serious | Moderate | Moderate | Serious |
| Font | Serious | Low | Serious | Serious | Serious | Serious | Serious | Serious |
| Narita | Serious | Low | Low | Serious | Moderate | Moderate | Serious | Serious |
| Pavlov | Serious | Low | Low | Low | Moderate | Serious | Moderate | Serious |
| Petrut | Serious | Low | Serious | Low | Moderate | Serious | Serious | Serious |
| Shen | Serious | Low | Low | Low | Serious | Serious | Moderate | Serious |
| Tuna | Serious | Low | Low | Low | Moderate | Moderate | Moderate | Serious |
| Wang | Serious | Low | Serious | Serious | Serious | Moderate | Serious | Serious |
| Yeaman | Serious | Low | Low | Low | Serious | Serious | Serious | Serious |

#### Supplementary figure 2: Risk of bias scoring


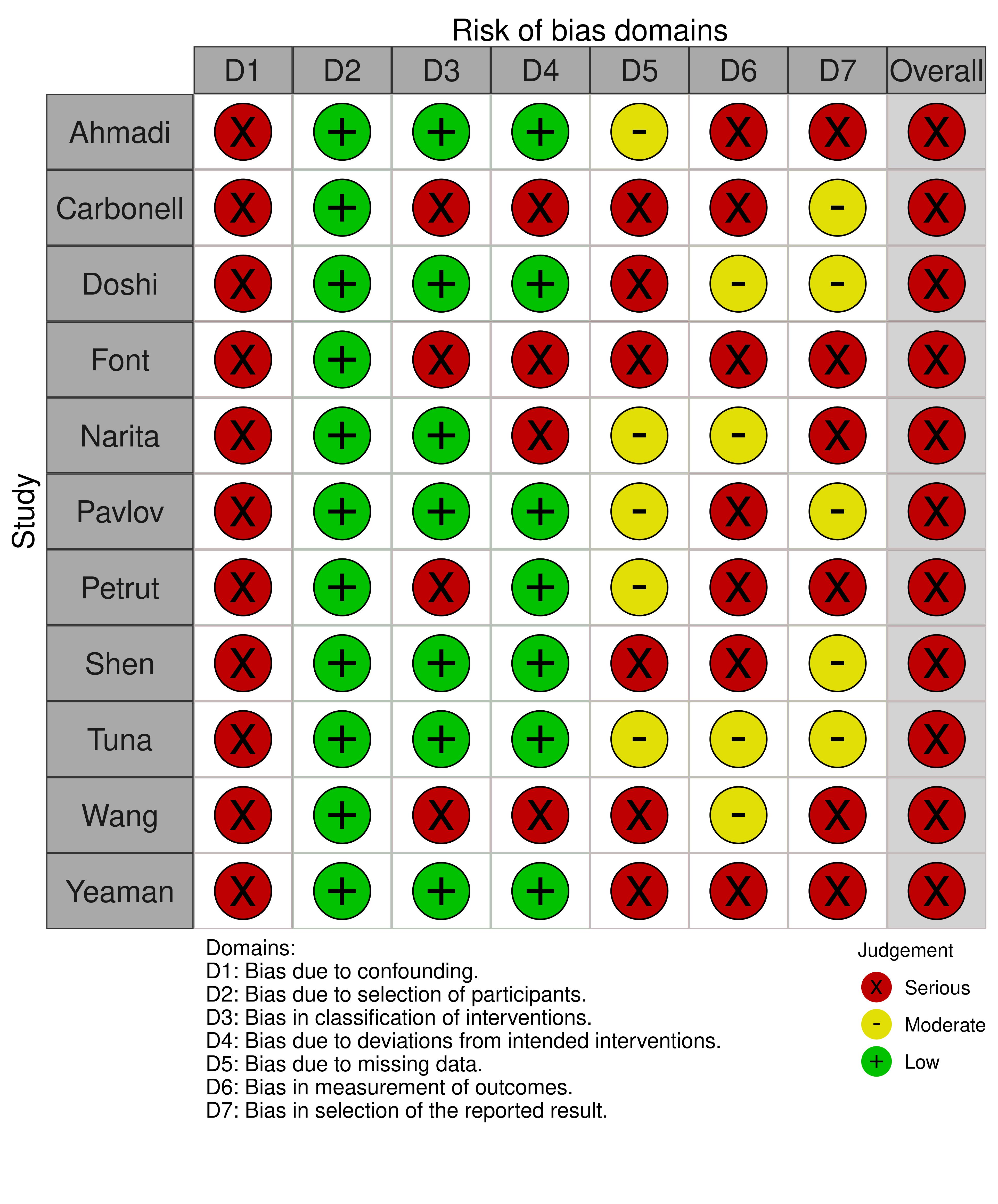

Supplement: Supplementary file 1 — Data S1. Methods. Table S1. Study characteristics. Table S2. Risk of bias assessment. Fig. S1. Ureter resection lengths. Fig. S2. Risk of bias scoring. [file BJU-135-908-s001.docx]
